# Rationale and Design for the LOnger-term effects of SARS-CoV-2 INfection on blood Vessels And blood pRessure (LOCHINVAR): an observational phenotyping study

**DOI:** 10.1136/openhrt-2022-002057

**Published:** 2022-06-23

**Authors:** Stefanie Lip, Linsay Mccallum, Christian Delles, John D McClure, Tomasz Guzik, Colin Berry, Rhian Touyz, Sandosh Padmanabhan

**Affiliations:** 1 BHF Glasgow Cardiovascular Research Centre, University of Glasgow, Glasgow, UK; 2 Queen Elizabeth University Hospital Campus, Glasgow, UK; 3 Research Institute of the McGill University Health Centre, McGill University, Montreal, Québec, Canada

**Keywords:** hypertension, COVID-19, biomarkers, research design

## Abstract

**Introduction:**

COVID-19 may lead to long-term endothelial consequences including hypertension, stroke and myocardial infarction. A pilot study ‘COVID-19 blood pressure endothelium interaction study’, which found that patients with normal blood pressure (BP) at the time of hospital admission with COVID-19 showed an 8.6 mm Hg higher BP ≥12 weeks after recovery, compared with a group without COVID-19. The ‘LOnger-term effects of SARS-CoV-2 INfection on blood Vessels And blood pRessure’(LOCHINVAR) study is designed to provide definitive evidence of the long-term impact of COVID-19 on BP.

**Methods and analysis:**

The LOCHINVAR study is an observational clinical phenotyping study comparing longitudinal BP change between individuals with and without COVID-19 infection. 150 participants (30–60 years) with no history of hypertension and not on BP lowering medications will be recruited to the study to attend three visits (baseline, 12 months, 18 months). Cases will be patients who were admitted to the Queen Elizabeth University Hospital (QEUH), Glasgow, UK, with suspected/confirmed COVID-19 until 31 December 2021 and who were alive at discharge. Controls will be those who have never had confirmed COVID-19 infection. All participants will undergo clinical and vascular phenotyping studies which will include 24-hour ambulatory BP monitoring systolic BP (ABPM SBP), brachial flow-mediated dilatation urine and blood samples to assess the renin-angiotensin system, vascular inflammation and immune status. The primary outcome is the change in systolic 24-hour ABPM (ABPM SBP) between the cases and controls. Sample size was calculated to detect a mean difference of 5 mm Hg ABPM SBP at 80% power.

**Ethics and dissemination:**

The protocol of this study has been approved by the West of Scotland Research Ethics Committee 5 (21/WS/0075), Scotland, UK. Written informed consent will be provided by all study participants. Study findings will be submitted to international peer-reviewed hypertension journals and will be presented at international scientific meetings.

**Trial registration number:**

NCT05087290.

**What is already known on this topic**

- COVID-19 may lead to long term endothelium dysfunction however the evidence of this is limited

**What this study adds**

- The LOCHINVAR study is an 18-month follow-up study of non-hypertensive adults who have recovered from COVID-19 and those with no history of COVID-19. It will determine if there is a sustained increase in blood pressure beyond 12 months post-COVID-19 and whether this is related to endothelial dysfunction or perturbations of the renin-angiotensin system.

**How this study might affect research, practice and/or policy**

- This study will help generate crucial evidence on the long-term impact of COVID-19 on blood pressure and will facilitate the prioritisation of targetable molecular pathways that can progress into potential therapies and clinical trials.

## Introduction

COVID-19 is an acute respiratory disease caused by the SARS-CoV-2 virus and declared a pandemic on 11 March 2020 by the WHO.^1^ SARS-CoV-2 enters target host cells through the interaction of the spike glycoprotein (S) with ACE2 on the target cell membrane serving as the SARS-CoV-2 receptor.^2^ ACE2 is a component of the renin-angiotensin system (RAS) and catalyses the conversion of Ang II to Ang-(1-7), a vasoprotective Ang II-derived peptide and plays an important role in blood pressure (BP) regulation.^3^ ACE2 is found in type 2 pneumocytes, macrophages, perivascular pericytes, cardiomyocytes and possibly endothelial cells, indicating the possible routes of infection by SARS-CoV-2,^2^ and potential consequences beyond the lungs especially the cardiovascular system^3^ leading to myocardial and endothelial dysfunction.^3^ Cardiovascular consequences are likely to be induced by severe systemic inflammatory cytokine storm, in patients with SARS-CoV2 requiring hospitalisation.^3^ Cardiac dysrhythmia, cardiac injury, myocarditis, heart failure, pulmonary embolism and disseminated intravascular coagulation are reported complications of COVID-19 lending credence to the extrapulmonary impact of COVID-19.^3^ Furthermore, epidemiological data suggest those at high cardiovascular risk (hypertension, obesity, diabetes) or those with prevalent cardiovascular disease are at higher risk of severe COVID-19.^4^ It is unclear if antihypertensive drugs that block the RAS (ACE inhibitors or angiotensin receptor blockers) alter prognosis in those with COVID-19 infection.^3^

Prior studies on the relationship between COVID-19 and the cardiovascular system are limited by case selection and retrospective data collection. A prospective pilot study ‘COVID-19 BP endothelium interaction study’ (ClinicalTrials.gov NCT04409847, funded by the Chief Scientific Office)^5^ compared 12-week postdischarge ambulatory BP measurements in non-hypertensive patients discharged from hospital after an admission with COVID-19 or a non-COVID diagnosis. This study showed that non-hypertensive COVID-19 patients had an 8.6 mm Hg higher 24-hour systolic BP (SBP) at 12 weeks postdischarge compared with those that did not have COVID-19 infection. A definitive prospective study with longer follow-up is required to confirm if BP is higher in post-COVID-19 patients and whether the higher BP is sustained over a longer period. The LOnger-term effects of SARS-CoV-2 INfection on blood Vessels And blood pRessure (LOCHINVAR) study is designed to answer this question along with pathophysiological studies through deep vascular phenotyping and biomarker studies of endothelial function and vascular injury. The LOCHINVAR study will help generate crucial evidence on the long-term impact of COVID-19 on BP and with information from our mechanistic studies which will facilitate the prioritisation of targetable molecular pathways that can rapidly progress into potential therapies and clinical trials.^6^

## Methods and analysis

### Study design

The LOCHINVAR study is a single-centre clinical study designed to determine the long-term impact of COVID-19 on BP. This study is a follow-up of the pilot COVID-19 blood pressure endothelium interaction study (OBELIX) study (ClinicalTrials.gov NCT04409847) which showed increased ambulatory BP monitoring (ABPM) SBP in COVID-19 patients postrecovery compared with contemporaneous non-COVID controls.

### Study participants, screening and recruitment

**Figure 1:**
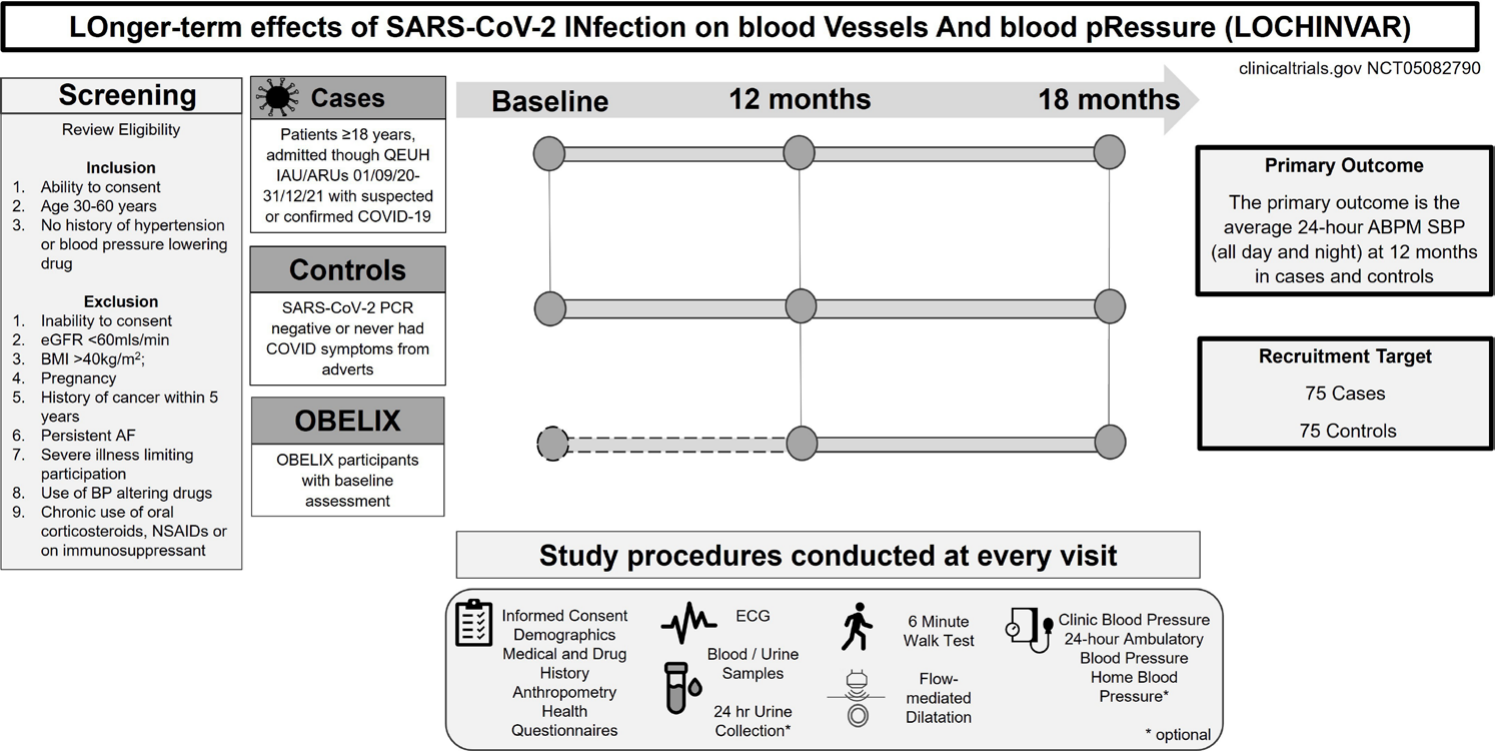
Study design, inclusion and exclusion criteria and study procedures. ABPM, ambulatory blood pressure monitoring; AF, atrial fibrillation; BMI, body mass index; BP, blood pressure; eGFR, estimated glomerular filtration rate; IAU, Immediate Admissions Unit. NSAID, non-steroidal anti-inflammatory drugs; OBELIX, COVID-19 blood pressure endothelium interaction study; QEUH, Queen Elizabeth University Hospital Glasgow; SBP, systolic blood pressure.

The study design, study visit schedule and inclusion/exclusion criteria is shown in figure 1. One hundred and fifty participants aged between 30 and 60 years in the National Health Service (NHS) Greater Glasgow and Clyde health board catchment area, will be invited to take part in the study to attend three study visits (baseline, 12 months and 18 months). Patients, who do not have a history of high BP or hypertension treatment, admitted through the QEUH immediate assessment unit and acute receiving units with suspected/confirmed COVID-19 between 1 September 2020 and 31 December 2021 will be invited after at least 12 weeks postdischarge. The clinical criteria for clinically suspected COVID-19 include either fever, new-onset cough and/or anosmia/dysgeusia and/or ≥2 of the following presenting features: fatigue/generalised weakness, headache, myalgia, sore throat/coryzal symptoms, breathlessness, anorexia/nausea/vomiting, diarrhoea, contact with known COVID-19 positive case. Patients who are SARS-CoV-2 RT-PCR positive and/or have diagnostic chest X-ray or CT features of COVID-19 will be classified as cases.

Control participants will be recruited from hospital admission records or through advertisement. Control patients recruited from hospital admission records will have an admission with COVID-19 like symptoms but are SARS-CoV-2 RT-PCR negative and have chest X-ray or CT (if performed during the relevant admission) that show low probability of COVID-19 and are SARS-CoV-2 IgG antibody negative at enrolment.

Controls recruited through advertising should have had no history of SARS-CoV-2 infection or have had a negative RT-PCR test previously and should have a negative SARS-CoV-2 IgG antibody test at enrolment. Controls will be matched for age, sex and diabetes status with cases by review of demographics of recruited cases and controls at 3 monthly intervals.

OBELIX participants have consented to be recontacted and will be invited to take part in the 12-month and 18-month follow-up visits in the LOCHINVAR study.

Screening for eligibility of all potential participants will be carried out by a member of the study team through review the electronic health record and electronic prescribing records or questionnaire responses. All participants identified as potentially eligible based on the inclusion criteria will be given a participant information sheet for the study containing contact details for the study team and given sufficient time to consider taking part. If the participant is interested, they will contact a member of the study team to discuss further and if they confirm interest in participating in the study, they will provide written, informed consent. Participants may withdraw from the study at any time.

### Study procedures

At each study visit, participants will undergo a set of clinical assessments which will comprise measurement of height, weight, waist circumference, office BP, oxygen saturations and heart rate. Body mass index (BMI) will be calculated from weight and height. An ECG will be performed. A manual of procedures has been collated for the study procedures below and kept in the study site file.

### Clinic BP

Clinic BP will be measured as per the British and Irish Hypertension Society (BIHS) guidelines using a validated BP monitor and an appropriate cuff based on the brachial arm circumference.^7^ The patient will be seated for at least 5 min, relaxed and not moving or speaking. BP will be measured in both arms, and the arm with the highest BP is selected. The measurement will be repeated a further two times, at least 1 min apart and the mean of readings two and three will be used. The appropriate cuff size will be used depending on the brachial arm circumference.

### Ambulatory BP monitoring

ABPM using Spacelabs 90217RM over 24 hours will be measured at each study visit according to BIHS guidelines.^7^ Participants will undergo ABPM for 24 hours with daytime readings every 30 min (0700–2159) and night-time readings every 60 min (2200–0659). The ABPM report is considered valid if there are 14 daytime measurements obtained. An appropriate cuff size will be used depending on the brachial arm circumference.

### Brachial flow-mediated dilation

At each study visit brachial flow-mediated dilation (FMD) will be measured using the UNEX EF38G device (UNEX Corporation, Nagoya, Japan) as previously described and based on the Glasgow Clinical Research Facility Standard Operating Procedures.^8^ In brief, the participant lies supine with their arm outstretched. A BP cuff is fitted on the forearm and the ultrasound probe is placed 5–10 cm proximal to the elbow using the probe holder. The brachial artery is identified in the short axis using the ultrasound probe. The automated tracking system activated to adjust the probe position. A baseline reading is obtained for 3 min. The occlusion cuff is then inflated 50 mm Hg above SBP for 5 min. At the end of this 5 min period, the maximum diameter is measured automatically, and the cuff is deflated and the brachial artery is tracked for 2 min. Automated outputs for rest diameter, maximum diameter and %FMD will be recorded. Additional manual quality checks and analysis were performed by a single operator (SL) where required. The %FMD is calculated as [(Max Diameter-Rest Diameter)/Rest Diameter]×100

### 6 mi walk test

The participant will sit for 10 min after which they will be asked to walk as long as possible between two markers for a maximum of 6 min to assess their exercise tolerance supervised by the research team. Level of shortness of breath and fatigue using the Borg scale, BP, oxygen saturations and heart rate will be measured pre and post 6 min walk test. This procedure has been modified from the American Thoracic Society and the Australian Lung Foundation/Australian Physiotherapy Association guidelines.^9 10^

### Questionnaires (Patient Health Questionnaire-9 and Euroqol 5-Dimension 3-Level)

The participant will be asked to complete the Patient Health Questionaire-9^11^ and Euroqol 5-Dimension 3-Level.^12^

### Blood samples

Blood sampling through venepuncture will be performed with samples collected in appropriate containers as described in table 1. Routine clinical biochemistry and haematology samples are sent to the local NHS laboratory. Samples taken for RAS fingerprinting (Attoquant Diagnostics, Vienna, Austria) and future biomarkers are centrifuged and serum is separated into 1 mL aliquots (nine aliquots in total) and then stored in a −80°C freezer. Immunotyping samples will be sent on ice to the BHF Glasgow Cardiovascular Research Centre for subsequent cytokine assessments as well as cellular immunity flow cytometry assays. All samples will be stored in a linked anonymised form.

**Table 1:** Blood samples

Routine clinical laboratory sampling	Full blood count, urea and electrolytes, bone profile, liver function tests, glucose, HBA1c, lipid profile, ferritin, B_12_, folate, coagulation screen, CRP
Additional clinical laboratory sampling	NTpro-BNP, D Dimer, hsTnI, renin, aldosterone, RAS fingerprinting
Urine sampling	24-hour urine collection for electrolytes (optional)
Vascular phenotyping	24-hour ABPM, ECG, brachial flow-mediated dilation (FMD), HBPM
SARS-CoV-2 antibody test	IgG antibody testing
Vascular and immune biomarkers (may be stored)	Vascular biomarkers Microparticles, oxidative stress markers (TBARS, 8-OHDG, nitrotyrosine), total antioxidant status, inflammatory biomarkers/adhesion molecules (ILs, MCP1, TNF, IFNs, sVCAM-1, sICAM, s-Selectin), RAS fingerprint ACE2 (s-ACE2, ACE2 activity), Ang peptides Immune biomarkers Trucount panel (CD3, CD4, CD8, CD14, CD19, CD45, CD56, HLA-DR), T cells (CD3, CD4, CD8, CD16, CD27, CD45RA, CD56, CD62L, TcRα/β TcRγ/δ), T cells II (CD3, CD4, CD8, CD31, CXCR4, CD27, CD45RA, CD56, CD40L), T cells III (CD3, CD4, CD45RA, CXCR5, CXCR, CCR6, CCR7, ICOS, PD-1), regulatory T cells (CD3, CD4, CD8, TcRα/β, CD25, CD45RA, CD127, FoxP3 (i/c)), B cells (CD5, CD10, CD19, CD24, CD27, CD38, CD86, IgD, IgM), Monocytes (CD4, CD14, CD16, CD19, CD45, HLA-DR), Dendritic cells (CD1c, CD16, CD11c,CD3/CD14/CD15/CD56, CD123, CD141, HLA-DR, CD80, SLAN) Future biomarker studies Blood and urine to be stored for future ethically approved biomarker studies.

ABPM, ambulatory blood pressure monitoring; CRP, C reactive protein; HBA1c, glycated hemoglobin; HBPM, home blood pressure monitoring; NTpro-BNP, N-terminal pro B-type natriuretic peptide; RAS, renin–angiotensin system.

### Urine sample and 24-hour urine collection

Participants will provide 10 mL of urine for a dipstick test and to be stored as 1 mL aliquots (three in total) in a −80°C freezer. A 24-hour cannister and an instructions sheet will be provided to participants to perform a 24-hour urine collection. The volume of 24-hour urine collected will be measured and 10 mls from the 24-hour urine sample will be aliquoted for storage while the rest will be processed in the local NHS laboratory for measurement of urine electrolytes.

### Optional study procedures

#### Home BP Monitoring

Home BP monitoring (HBPM) will be performed using OMRON M3 device in accordance with BIHS guidelines.^7^ Readings will be recorded, in triplicate, morning (0600–1200) and evening (1800–0000) for 7 days. The first day’s readings will be excluded from the mean if there are major discrepancies between the first and second reading. A minimum of five sets of morning and evening readings will be required for HBPM to be valid within a 10-day period. HBPM will be recommended for all participants if ABPM shows elevated BP.

### Adverse events

No specific adverse events are anticipated other than participant discomfort related to venepuncture, ABPM and FMD.

### Poststudy care

At each study visit, the participant will be informed results of investigations, and clinically important information will be communicated to the participant’s general practitioner.

### Study objectives and statistical analysis

#### Primary outcome

The primary outcome is the average 24-hour ABPM SBP (all day and night) at 12 months in SARS-CoV-2 positive cases and in SARS-CoV-2 negative controls

#### Secondary outcomes

Secondary outcomes are the average of the following measures SARS-CoV-2 positive cases and in SARS-CoV-2 negative controls at 12 months and 18 months: 24-hour ABPM diastolic BP (DBP); day ABPM SBP; day ABPM DBP; night ABPM SBP; night ABPM DBP; 24-hour ABPM HR and 24-hour urine sodium

#### Tertiary outcomes

Tertiary outcomes are % FMD; Exercise tolerance—distance walked in 6 min; Quality of life and mood—difference in longitudinal changes in QoL and mood between groups; mean HBPM SBP; mean HBPM DBP; day HBPM SBP; evening HBPM SBP; day HBPM DBP and evening HBPM DBP at 12 months and 18 months.

### Sample size calculation

From the OBELIX pilot study, we have established that ABPM SBP is normally distributed with a SD of 10 mm Hg. A mean difference of 5 mm Hg ABPM SBP between groups was determined to be the minimum clinically relevant difference. We will require 64 COVID-19 cases and 64 non-COVID control subjects to reject the null hypothesis with an alpha=0.05% and 80% power. The study requires a total of 150 subjects to be recruited (128 completed subjects) after accounting for approximately 15% missing data and ABPM errors (typical for ABPM studies). OBELIX has recruited 48 subjects and these participants will be invited into LOCHINVAR. The LOCHINVAR study will recruit the remaining subjects.

### Statistical analysis

The primary analysis (primary outcome 24-hour ABPM SBP at 12 months) will use linear regression to compare 24-hour ABPM SBP between SARS-CoV-2 positive and SARS-CoV-2 negative subjects with adjustment for covariates including age, sex and BMI. The adjusted mean difference, with a 95% CI and associated p value, will be reported.

Secondary analyses will extend this regression model to examine the impact of baseline patient characteristics, and to investigate the possibility of interactions with COVID-19 clinical status. For longitudinal analysis we shall use linear mixed models as appropriate to compare changes in the mean 24-hour ABPM SBP between SARS-CoV-2 positive and SARS-CoV-2 negative subjects with adjustment for baseline 24-hour ABPM SBP and baseline covariates.

All statistical analyses will be performed using R Software V.4.0.2 or later.

### Study sponsorship, monitoring and audit

The study is sponsored by the NHS Greater Glasgow and Clyde and is coordinated in the Glasgow Clinical Research Facility. The study will be subject to audit by the Sponsor to ensure quality of study data and compliance with regulations. Any change in the study protocol will require an amendment which will be initiated by the chief investigator in discussion with the sponsor and will be ethically approved.

### Data collection, management and retention

All patients will be assigned a unique study identifier which will not contain any identifiable information. Patients in Scotland has a unique Community Health Index (CHI) number. Where linkage is required the key to CHI identification will be kept separate and only on an NHS computer. Data will be recoded in CASTOR EDC (www.castoredc.com) electronic case report forms (eCRF) held centrally on their ISO 27001 certified data management system. CASTOR EDC have been vendor assessed by the study sponsor NHSGGC in accordance with regulatory guidelines. CASTOR complies with all applicable laws and regulations, including ICH E6 Good Clinical Practice (GCP), 21 CFR Part 11, EU Annex 11, General Data Protection Regulation, HIPAA (US), ISO 9001 and ISO 27001. Data entry will be completed by either the study nurse, clinical fellow or an investigator. Data will be validated at the point of entry into the eCRF and can be checked by sponsor audit if required. To enable evaluations and/or audits from regulatory authorities, records will be kept, including the identity of all participating subjects (sufficient information to link records), all original signed informed consent forms, source documents, and detailed records of treatment disposition in accordance with ICH GCP and local regulations. All samples will be retained by the investigators. Data will be retained for a minimum of 20 years.

## Dissemination

An annual report will be issued to the Research Ethics Committee and to the funder (Heart Research UK).

Complete study results will be submitted for presentation at an international meeting, published in a peer-reviewed journal, and shared on social media. After publication, LOCHINVAR participants will be informed of results by letter. A lay summary will be made available to the participants who have taken part in the study and may be published on the funder, university or NHS website/newsletter.

## Data Availability

No data are available.
